# A Single Intramedullary K-Wire Is Sufficient for the Management of Nonthumb Metacarpal Shaft Fractures

**DOI:** 10.1155/2021/9963186

**Published:** 2021-05-04

**Authors:** Mohamed I. Abulsoud, Mohammed Elmarghany, Tharwat Abdelghany, Mohamed Abdelaal, Mohamed F. Elhalawany, Ahmed R. Zakaria

**Affiliations:** ^1^Department of Orthopedic Surgery, Faculty of Medicine, Al-Azhar University, Cairo, Egypt; ^2^Department of Orthopedic Surgery, Helwan University, Helwan, Egypt

## Abstract

**Objective:**

This study aims to evaluate the outcome after the internal fixation of diaphyseal metacarpal fractures by a single intramedullary K-wire.

**Methods:**

In this prospective case series study, conducted from July 2017 to June 2019 in 23 adult patients with a single, unstable, diaphyseal metacarpal fracture, outcomes after internal surgical fixation using a single antegrade intramedullary K-wire were evaluated. The outcomes were evaluated by union rate, time to union, handgrip measurements at 6 and 12 months, and the modified Disabilities of the Arm, Shoulder, and Hand (DASH) score at 12 months.

**Results:**

The study population consisted of 17 males and 6 females, with a mean patient age of 28.4 ± 8.5 years (range, 16–45 years). The median time to final follow-up was 14 ± 1.8 months (range: 12–24 months). The mean duration of the union was 7.3 ± 1.6 weeks (range: 5–11 weeks), with a union rate of 95.7% (22 cases). The mean handgrip strength was 68% ± 12.8% of the strength of the uninjured hand after 6 months and 92.7% ± 6.9% after 12 months. The mean modified DASH score was 2.6 ± 0.26 after 12 months (range: 0–5.8). There were no cases of malrotation or infection. In conclusion, using a single 1.8–2.0 mm K-wire gives excellent functional outcomes and union rate without significant complications when used to treat an unstable metacarpal shaft fracture.

## 1. Introduction

Metacarpal fractures are the third most common upper limb injury in young adults. when combined with phalangeal fractures, they are the most common upper limb injury [1, 2]. Men and young adults are more vulnerable to these injuries, as are people of low socioeconomic status [1]. The leading mechanisms of injury are direct trauma and sports trauma [1, 2]. Diaphyseal metacarpal fractures cause marked angulation and shortening, impeding the function of extensor and flexor tendons [3–5]. Even small degrees of malrotation are poorly tolerated, leading to digital overlap and impairments of hand functions [6], as the deep transverse metacarpal ligament helps in maintaining shortening and rotation [7]. Metacarpal fractures are more easily tolerated and can be treated nonoperatively if they occur more ulnarly and distally [8]. Surgical options for treatment show wide variabilities without a preference for the fixation method [9, 10].

As early as 1953, Vom [11] described intramedullary fixation of metacarpal fracture and introduced a K-wire through the head of the metacarpal. Foucher's [12] bouquet technique is the most popular approach for antegrade K-wire fixation; it was initially restricted to the neck of fifth metacarpal fractures, but has been applied to diaphyseal fractures with different modifications [13–17]. In surgical practice, ad hoc technological instruments (e.g., plates) often are preferred as opposed to K-wires because they are supposed to fix the fracture better. However, in adult upper limb fractures, a safe and effective fixation can be obtained with smooth wires and rods with very good functional outcome [18–20].

### 1.1. Specific Aim and Hypothesis

This study aims to evaluate outcomes after internal fixation of diaphyseal metacarpal fractures using a single intramedullary K-wire.

We hypothesized that a single intramedullary K-wire is enough to fixate a displaced metacarpal fracture, leading to full union and a satisfactory outcome without major complications.

## 2. Methods

A case series study was conducted to evaluate the outcome of single antegrade intramedullary K-wire fixation on displaced metacarpal fractures within 2 weeks of the initial injury. This study included 23 consecutive patients treated from July 2017 to June 2019.

To be included, a patient had to be an adult older than 16 years with a single unstable diaphyseal fracture of the metacarpal. Unstable fractures were defined as having angulation >40°, shortening >2 mm, or malrotation.

Cases were excluded from the study if the patient had an open fracture, associated compartment syndrome of the hand, intraarticular extension, multiple metacarpal fractures, or severe comminution (AO/OTA types 77. 3.2C2 and 77. 3.2C3), or if the patient was <16 years.

**Figure 1:**
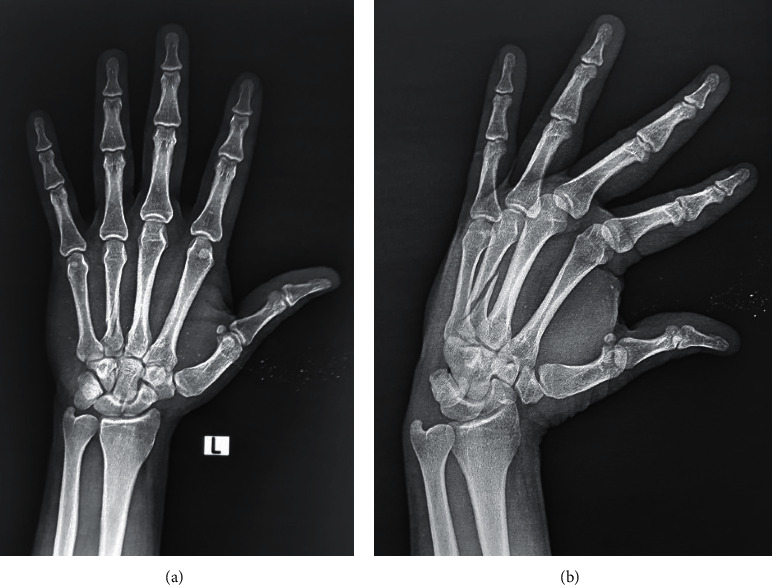
A 30-year-old male patient with spiral fracture at the fourth metacarpal: the medulla of the second and third metacarpal bones is too narrow, while the medulla of the fourth metacarpal is patent which allows intramedullary fixation.

All patients received a thorough clinical evaluation that included general and local examinations and X-rays from two different views to ensure there were no other fractures and to ensure patency of the medullary canal (Figure 1). All cases were treated with internal fixation by a single antegrade intramedullary K-wire.

The STROBE guidelines for cohort studies have been followed.

All patients gave consent for participation in the study. The study was approved by the institutional ethics committee.

### 2.1. Surgical Technique

All surgeries were performed under general anesthesia, used fluoroscopic control, did not use a tourniquet, and had an antibiotic (1 g cefotaxime) administered while inducing anesthesia.

**Figure 2:**
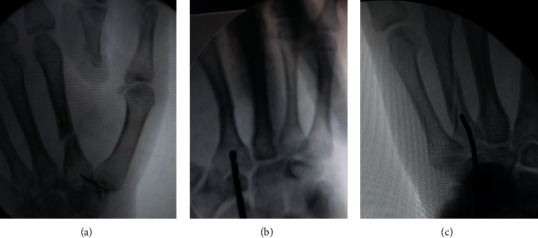
(a) Fluoroscopic photo showing the identification of the incision site by a syringe needle. (b) Fluoroscopic photo for antegrade fixation of a fracture of the second metacarpal shaft. A 2.5 drill bit is used for drilling of the dorsal cortex. (c) Fluoroscopic photo shows the advancement of the blunt-tipped prebent K-wire through the entry hole in the dorsal cortex.

After adequate disinfection of the skin and draping, the patient's hand was positioned on a radiolucent table. The base of the metacarpal was determined with a syringe needle to avoid an inappropriate incision (Figure 2(a)). A 2-3 cm skin incision was made on the dorsal side of the base of the involved metacarpal, allowing for good visualization of the base of the metacarpal as an entry point. The surgeon dissected the subcutaneous tissue and identified the extensor tendons, protecting them throughout the procedure by retracting them ulnarly.

With a sleeve in place in the center of the base of the dorsum to protect the extensor tendon of the involved metacarpal, a 2.5 mm drill bit was used to open the dorsal cortex at an angle of about 45° cranially while taking care not to violate the volar cortex (Figure 2(b)).

After cutting the trocar tip of a prebent 1.8–2.0 mm K-wire, a T-handle device was used to introduce the wire inside the metacarpal shaft (Figure 2(c)).

**Figure 3:**
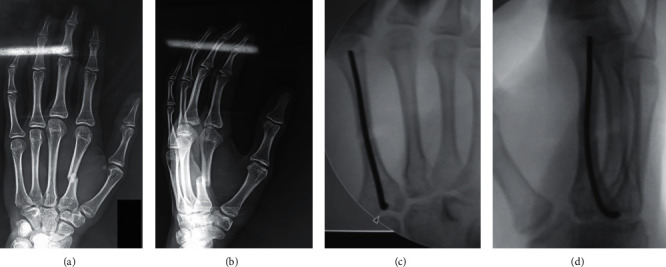
A 17-year-old male patient whose X-rays of anteroposterior and oblique views of the hand show a displaced diaphyseal fracture of the second metacarpal. Fluoroscopic photos show the final fixation of the displaced second metacarpal fracture with the three-point fixation of the intramedullary K-wire.

Next, the rotation was assessed clinically and radiographically, with adjustments made until any malrotation was addressed and appropriate reduction had been achieved. During this process, the K-wire was advanced into the distal segment until it reached the metacarpal head where it was adjusted to achieve the principle of three-point fixation. Violation of the articular surface of the metacarpophalangeal joint was carefully avoided. To allow skin closure, the K-wire was cut short proximally. To avoid any friction, which could lead to tendon rupture or could limit the range of motion in the finger, the bend in the K-wire was positioned away from the track of the extensor tendon (Figure 3).

The skin incision was closed with simple stitches, and a splint was applied below the elbow for 2 weeks.

### 2.2. Postoperative Program

Patients visited the outpatient clinic 2 weeks postoperatively to have the stitches and splint removed. X-rays were taken to ensure adequate reduction and fixation. The patient was encouraged to move all joints of the hand actively and passively. Regular follow-up visits were scheduled until full union had been achieved.

### 2.3. Hardware Removal

**Figure 4:**
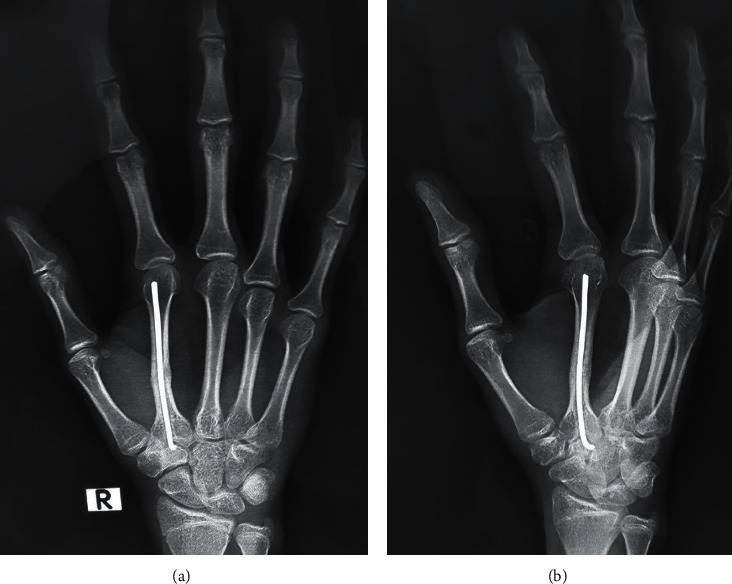
X-ray showing the 6-month follow-up of the patient, prior to K-wire removal.

**Figure 5:**
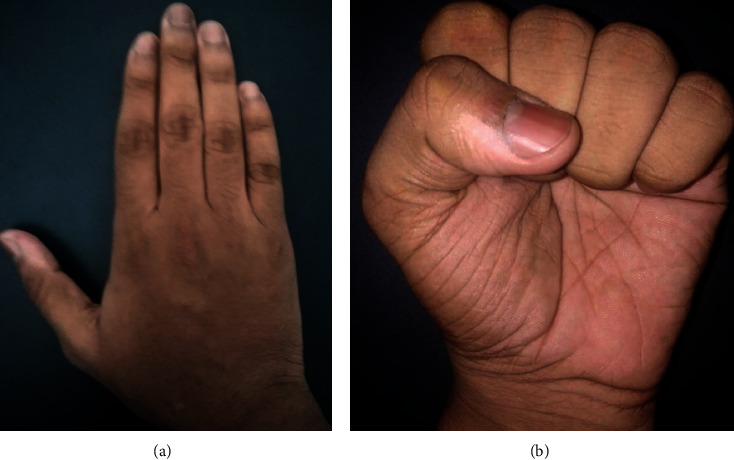
Functional outcome after K-wire removal with full range of motion and excellent functional outcome.

The K-wires were removed between 3 and 12 months postoperatively. Removals were performed under either general or local anesthesia after the anesthesia team discussed both options with the patient (Figures 4 and 5).

### 2.4. Statistical Analysis

Data were analyzed using Statistical Program for Social Science (SPSS), version 15.0 (SPSS Inc., Chicago, Illinois). Quantitative data were expressed as means ± standard deviations after confirmation of normal distribution. Data that were not distributed normally were expressed as medians and interquartile ranges. Qualitative data were expressed as frequencies and percentages. *P* value <0.05 was statistically significant.

## 3. Results

The study treated 23 metacarpal fractures in 23 patients (17 males and 6 females). All fractures were closed, unstable single fractures.

There were eight patients with a fractured second metacarpal, three with a fractured third metacarpal, four with a fractured fourth metacarpal, and eight with a fractured fifth metacarpal (Table 1).

The mean patient age was 28.4 ± 8.5 years (range: 16–45 years). The median time to final follow-up was 14 ± 1.8 months (range: 12–24 months).

The mean time to union was 7.3 ± 1.6 weeks (range: 5–11 weeks), with a union rate of 95.7% (22 cases). Only one patient failed to develop union, due to the use of a small-diameter K-wire. This patient was subsequently treated by open reduction and internal fixation using a plate and screw and an autologous bone graft.

At 6 and 12 months postoperatively, each patients' handgrip was assessed using the CAMRY Digital Hand Dynamometer Grip Strength Measurement, measuring capacity of 198 lbs/90 kgs by comparing the injured and uninjured hands. The mean handgrip strength of the injured hand was 68% ± 12.8% of the strength of the uninjured hand after 6 months and 92.7% ± 6.9% of the strength of the uninjured hand after 12 months.

The functional outcome was assessed according to the modified Disabilities of the Arm, Shoulder, and Hand (DASH) score, with scores ranging from 0 (best possible score) to 100 (worst possible score). The DASH score measures the severity of symptoms, including pain, stiffness, weakness, and tingling, as well as the ability to perform activities of daily living, including opening a jar, turning a key, writing, pushing a door, washing, dressing, and completing household tasks. The mean score was 2.6 ± 0.26 after 12 months (range: 0–5.8; Table 2).

Three patients (13%) developed stiffness of the interphalangeal joint due to not completing hand exercises at home. These patients received physical therapy and improved by the end of the follow-up.

None of our patients developed malrotation or wound infection. There were two patients (8.6%) who developed joint penetration of the metacarpophalangeal joint during follow-up, although this did not affect the outcome (Table 3).

## 4. Discussion

The study shows that a single antegrade K-wire can be used to treat an unstable metacarpal shaft fracture, with excellent functional outcomes and a low complication rate.

Although plate fixation is an attractive option in the treatment of metacarpal shaft fractures due to its stable fixation and biomechanical stability [21], it has a relatively high complication rate of up to one-third of cases [22]. In a study, plate fixation resulted in functional impairments that required secondary surgery in 17% of cases [23]. Even in a study using modern, low-profile plates, various complications led to plate removal in 40% of cases within 9.6 months after surgery [24].

To the best of our knowledge, this is the first study to describe and investigate outcomes of fixation of midshaft metacarpal fractures using a single, buried K-wire. To reduce the confounders, multiple metacarpal fractures, metacarpal neck fractures, and highly comminuted fractures were excluded from the study. In 23 cases with strict inclusion criteria, the functional outcomes were excellent. The mean modified DASH score was 2.6 ± 0.26 at 12 months postoperatively, and the mean handgrip strength was 68% ± 12.8% after 6 months and 92.7% ± 6.9% after 12 months. These outcomes are comparable to those of most other studies treating such fractures.

The union rate was excellent (95.7%). The one case of nonunion was due to the use of a thin K-wire (1.2 mm), so we recommend using 1.8–2 mm K-wires. No cases of clinical malrotation were reported in our study. This indicates that insertion of a single intramedullary K-wire with the use of a splint for 2 weeks can maintain rotational stability in fracture types 77. 3.2A and 77. 3.2B.

In a recent study using CT to measure the diameter of the nonthumb metacarpal shaft, the narrowest point of the medullary canal was found to be between 2.6 and 3.7 mm [25], supporting the observations from our study that the use of a single intramedullary K-wire with a diameter up to 2 mm gives very good stability.

No cases of infection were detected in this study of the buried K-wire technique, although with an exposed K-wire, the infection rate is about 6% [26]. This is in line with results published by Ridley and colleagues [27] showing that the risk of infection is higher in exposed K-wires than in buried K-wires, especially in the treatment of metacarpal fractures.

The percutaneous antegrade intramedullary fixation has been described by Landi et al. [18]. The use of the blunt tip of the K-wire has been described previously by Rocchi et al. [28]. Their large sample included single and multiple K-wires and cases with both shaft and neck fractures but obtained excellent results with minimal complications. However, both techniques used unburied K-wires without focusing on the use of a single K-wire.

Although two cases of metacarpophalangeal joint penetration were observed during follow-up, the final functional outcome was not affected.

Despite the short immobilization time in our study (2 weeks), three patients reported stiffness in the corresponding interphalangeal joint during follow-up. These symptoms were improved by physiotherapy, and the patients had no limitation of motion at the final follow-up. Note that these cases of stiffness and the joint penetration cases were in different patients.

Various retrograde and antegrade techniques have been described over 70 years for intramedullary K-wire fixation of metacarpal fractures, but no technique has been proven to be definitively superior [29]. A biomechanical study concluded that using a single 1.6 mm K-wire results in significantly more stiffness than three 0.8 K-wires [30]. Smooth and unlocked fixation devices are not out of date, but they should be used in the right way. The recent literature continues to prove it. The three-point intramedullary fixation system could be superior to the rigid interfragmentary fixation and it does not hinder the movement [19, 20].

However, the study has some limitations. First, it lacks a comparison group using other techniques. Second, it required a second procedure to remove the K-wire, although most of the patients did not report major complaints during follow-up. Finally, all patients in the study were young and healthy, and the validity of this technique needs to be tested in an older age group and those with osteoporosis.

In conclusion, the use of a single 1.8–2.0 mm K-wire and immobilization for 2 weeks to treat a displaced metacarpal shaft fracture results in excellent functional outcomes and an excellent union rate without significant complications. The technique should be further validated in cases of multiple fractures or open fractures but should be used with caution in cases of osteoporotic fractures.

## Figures and Tables

**Table 1:** Demographic data.

Characteristics	Value
*Age (years)*	
Minimum	16
Maximum	45
Mean (SD)	28.4 8.5
*Gender*	
Male	19
Female	6
*Involved bone*	
Second metacarpal	8
Third metacarpal	4
Fourth metacarpal	3
Fifth metacarpal	8

**Table 2:** Results.

Characteristics	Value
*Time to union (weeks)*	
Minimum	5
Maximum	11
Mean (SD)	7.3 ± 1.6
Union rate	22/23 (95.7%)
Handgrip strength (6 m)	19
Minimum	6
*Maximum*	
Mean (SD)	68 ± 12.8%
*Handgrip strength (12 m)*	
Minimum	3
Maximum	8
Mean (SD)	92.7 ± 6.9%
*DASH score (12 m)*	
Minimum	0
Maximum	5.8
Mean (SD)	2.6 ± 0.26

**Table 3:** Complications.

Nonunion	Stiffness	MPJ penetration
1 (4.3%)	3 (13%)	2 (8.6%)

## Data Availability

The datasets used and analyzed during the current study are available from the corresponding author on request.
